# Strategies to Improve Meat Products’ Quality

**DOI:** 10.3390/foods9121883

**Published:** 2020-12-17

**Authors:** Claudiu Ștefan Ursachi, Simona Perța-Crișan, Florentina-Daniela Munteanu

**Affiliations:** Faculty of Food Engineering, Tourism and Environmental Protection, “Aurel Vlaicu” University of Arad, 310330 Arad, Romania; claudiu.ursachi@uav.ro (C.Ș.U.); simona.perta-crisan@uav.ro (S.P.-C.)

**Keywords:** meat products quality, antioxidants, dietary fibers, probiotics, processing technologies

## Abstract

Meat products represent an important component of the human diet, their consumption registering a global increase over the last few years. These foodstuffs constitute a good source of energy and some nutrients, such as essential amino acids, high biological value proteins, minerals like iron, zinc, selenium, manganese and B-complex vitamins, especially vitamin B12. On the other hand, nutritionists have associated high consumption of processed meat with an increased risk of several diseases. Researchers and processed meat producers are involved in finding methods to eliminate nutritional deficiencies and potentially toxic compounds, to obtain healthier products and at the same time with no affecting the sensorial quality and safety of the meat products. The present review aims to summarize the newest trends regarding the most important methods that can be applied to obtain high-quality products. Nutritional enrichment with natural bioactive plant compounds (antioxidants, dietary fibers) or probiotics, reduction of harmful components (salt, nitrate/nitrite, N-nitrosamines) and the use of alternative technologies (high-pressure processing, cold plasma, ultrasounds) are the most used current strategies to accomplish this aim.

## 1. Introduction

It is generally accepted that meat refers to the skeletal muscle and its associated tissues provided by animals, and in accordance to the European legislation it represents the edible parts removed from the carcass of domestic ungulates including porcine, bovine, ovine and caprine animals, domestic solipeds as well as poultry, lagomorphs, farmed game, small and large wild game [1].

Meat products are defined as processed products in which the fresh meat has been subjected to some processing procedures (e.g., dehydration, fermentation, curing, smoking, thermal preparation) so that the section of the product shows that the characteristics of fresh meat are completely absent [1,2].

The consumption of meat has been an important component of the human diet for a long time, and is considered essential for optimal development of the organism and also indispensable for the life of modern society, from the nutritional point of view [3,4]. Through this meaning, meat represents for the human diet an important source of energy and range of nutrients, including high-quality proteins (with a good balance of amino acids), minerals (iron, zinc, selenium, manganese) and vitamins (B12, folic acid). Regarding the nutritional composition of 100 g of different meat cut, these might contain 17.3 to 24.1 g of proteins, 0.3 to 2 µg B_12_ vitamins and minerals (24–77 mg of sodium, 145–221 mg of phosphorous, 0.6–2 mg of iron, and 0.9–2 mg of zinc), while the energetic value might be between 105–176 kcal [5].

A great number of studies indicate that, besides macronutrients, especially proteins and lipids, meat and meat products are also rich in some bioactive components with antioxidant properties that have an important role in the consumer’s health [6]. For example, recent studies confirmed the antioxidant properties of L-carnitine and L-carnosine by their radical scavenging activity and metal ions chelating ability. Other studies, performed on animals, reported that intake of these compounds contributed to a significant decrease in serum triglycerides and total cholesterol levels, and it also may prevent fatty liver [6]. Besides antioxidant properties of lipoic acid, literature sources show also their hypotensive and immunomodulatory effect [7]. Taurine was also found to protect the retina and reduce the level of free and esterified cholesterol. Meat extracted peptides exhibited antithrombotic properties and showed a great cytotoxic effect against different cancer cells [8]. On the other hand, despite nutritional benefits, some studies pointed out a connection between the high level of red meat consumption and the increase of risk for various types of cancer, especially colorectal ones [9]. Consumption of processed meat can also be associated with the risk of heart diseases and different metabolic disorders (diabetes, weight gain) [10,11]. Several mechanisms could underlie the association between meat intake and health risk. Considering that processed meat is rich in saturated fatty acids, salt, carcinogenic and mutagenic components can be generated during the processing stages, as well as N-nitroso compounds, biogenic amines, heterocyclic aromatic amines and polycyclic aromatic hydrocarbons [12,13,14]. For this reason, in the last few years, consumers have been claiming for nutritionally improved meat products with potential benefits to human health. To meet their demands, researchers have focused on the possibility of developing reformulated meat products with a lower content of some undesirable additives, fats, cholesterol or sodium chloride and with an improved composition of unsaturated fatty acids and other bioactive compounds [15,16].

**Figure 1:**
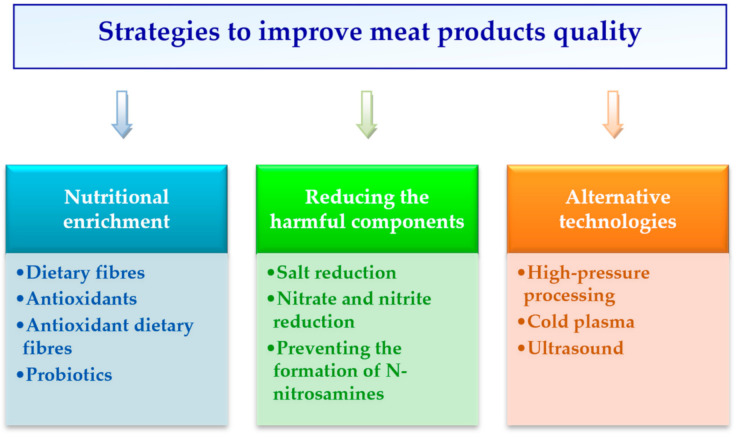
Proposed strategies for improving the quality of meat products.

As shown in Figure 1, the strategies used to enhance meat products’ quality are principally based on improving their composition by incorporating some bioactive components, reducing quantities of the exogenous additives and of indigenous noxious compounds that are formed, and application of alternative technologies.

## 2. Nutritional Enrichment

In the last few years, a strategy to improve meat product quality proved to be the incorporation of some functional or bioactive components. These compounds include dietary fibers, antioxidants, probiotics and prebiotics, which are increasing the nutritional value.

### 2.1. Dietary Fibres

The negative effects of some nutrients (saturated fatty acids, cholesterol and triglyceride) on human health [17,18] can be minimized by the addition of the dietary fibers to meat products, due to their physiological benefits. The European Food Safety Authority defines dietary fibers as non-digestible carbohydrates plus lignin, including all carbohydrate components occurring in foods that are non-digestible in the human small intestine and pass into the large intestine [19]. Dietary fibers can be classified by several criteria. According to their chemical properties, the main types of dietary fibers are non-starch polysaccharides (cellulose, hemicelluloses, pectins, hydrocolloids), resistant oligosaccharides (fructo-oligosaccharides, galacto-oligosaccharides, other resistant oligosaccharides), resistant starch and lignin [20]. According to their source, dietary fibers can be categorized into plant polysaccharides, animal polysaccharides and synthetic ones [21]. However, the most commonly used system of classification is the one based on their water solubility and fermentation behavior. Thus, dietary fibers are classified into insoluble dietary fibers and less-fermented fibers (cellulose, part of hemicelluloses and lignin) and soluble dietary fibers or well-fermented fibers (pectins, pentosans, gums and mucilages) [21]. Dietary fiber incorporation in meat products is nowadays in practice due to their nutritional, functional and technological values. There is a proved efficiency in their prevention of fatty acids and cholesterol absorption and reduction of obesity risk, cardiovascular diseases, colon cancer and different other disorders [17]. Moreover, fibers provide other health benefits, such as antidiabetic and antioxidative ones, and are stimulants of the organism’s immune system. The addition of dietary fibers to meat products can also enhance some technological properties, such as improving emulsion stability; increasing water-holding capacity of minced meats; decreasing losses on cooking; improving texture and rheological properties of meat products; and increasing the efficiency of the product [22,23]. Various types of dietary fibers used to improve the quality of meat products are presented in Table 1.

### 2.2. Antioxidants

Lipids, proteins or pigments oxidation represents one of the major causes for the quality deterioration of meat and meat products because it affects their color, taste, texture and nutritional value (e.g., losses of essential amino acids, essential fatty acids and vitamins). Furthermore, oxidative reactions cause the production of some potentially cytotoxic and genotoxic compounds, such as peroxy radicals, fatty acids peroxides, cholesterol hydroperoxide, malonaldehyde [4,31]. The rate of oxidation processes can be efficiently slowed by using antioxidants. On this line, synthetic antioxidants like butylated hydroxytoluene (BHT), butylated hydroxyanisole (BHA), propyl gallate (PG) or tertiary butylhydroquinone (TBHQ) have long been used in the meat industry to control oxidation reactions. In the last few years, synthetic antioxidants have been rejected by consumers for health reasons, which have forced the food industry to find alternative possibilities for controlling and reducing oxidative degradation of meat products. Therefore, the use of natural antioxidants derived from different vegetal materials can be considered a valid strategy to delay or inhibit lipids and proteins oxidation [32]. Antioxidant activity of natural sources is attributed to a wide range of chemical compounds, such as phenolic compounds (phenolic acids, phenolic diterpenes, flavonoids), volatile oils, carotenoids, vitamins (vitamin A, vitamin C and vitamin E) and bioactive peptides [4,17,33]. Vegetables constitute an important source of antioxidants which are present in all parts of the plant (roots, seeds, leaves, husks, flowers or fruits). Many studies proved the efficiency of natural antioxidants from herbs and spices (clove, rosemary, oregano, nutmeg, sage, cinnamon), green tea leaves, aloe vera, grapes, dark berries or citruses in oxidation prevention of meat products [34]. Besides improving shelf-life, oxidative stability and sensory qualities, these natural compounds may also add to meat products’ functional properties and health benefits [17]. Some types of natural antioxidants used in meat products are presented in Table 2.

### 2.3. Antioxidant Dietary Fibres

In 1998, Saura-Calixto introduced the concept of antioxidant dietary fiber (ADF) [44]. The characteristic of ADF is the significant content of both antioxidants and dietary fibers in a single material. The incorporation of ADF in meat mixture combines the physiological effects of these bioactive compounds, which not only delay lipid oxidation but also improve sensory properties and increase the nutritional value of meat products [45]. To be considered an ADF, an ingredient should possess a free radical scavenging activity equivalent to at least 50 mg of vitamin E; the ability to delay lipid oxidation equivalent to at least 200 mg of vitamin E; dietary fiber content higher than 50% reported to dry matter [44,46].

Besides cereals, legumes and algae, several fruits and vegetables are an important source of both antioxidants and dietary fibers [46]. The by-products of some vegetable products (peels, leaves, seeds, stems, pomace, etc.) meet the criteria of ADF and can be considered an important source of these bioactive components. Considering the nutritional value, cost-efficiency and added value of functional ingredients derived from plant by-products, they present a real interest both for researchers and food processors, for the development of new and healthier foods. Several studies showed the great potential of different plant by-products to be reconsidered as an important and cheaper source of ADFs and not as a waste. Thus, the apple pomace and peel [47], red grape pomace [48], cabbage powder [49], mango peel powder [50], guava peel [51], blueberry pomace powder [52], cocoa bean shell [53], pineapple pomace [54], coffee husk [55] and spent coffee grounds [56,57], are reported as sources with a high content of dietary fibers and antioxidant compounds. Table 3 summarizes the main advantages of the addition of ADFs to different meat products.

### 2.4. Probiotics

According to Food and Agriculture Organization of the United Nations (FAO) and World Health Organization (WHO) and adopted by the International Scientific Association for Probiotics and Prebiotics (ISAPP), probiotics refer to non-pathogenic living microorganisms which, when present in adequate amounts, confer health benefits to the host [66,67]. Probiotic functional food products have known a great development in the last few years and can be considered the future of health-promoting foods [68]. Several researchers considered that the consumption of probiotics provides a variety of health benefits, including regulation of intestinal transit, normalization of perturbed microbiota and maintenance of intestinal barrier integrity [69,70]. Different species of probiotics can also increase enterocyte turnover, colonization resistance, short-chain fatty acids production, and competitive exclusion of pathogens [71,72]. The main species of microorganisms used as probiotics in food products are *Lactobacillus* and *Bifidobacterium* but have also been reported a lot of other species of bacteria *(Lactococcus, Enterococcus, Propionibacterium)* or yeasts (*Saccharomyces)* that are also available [73]. The principal probiotic microorganisms with claimed health benefits for humans are summarized in Table 4.

Probiotic meat products represent a part of the functional group of foods which contain probiotic microorganisms. For being considered probiotic, the selected microbial culture should be able to tolerate gastrointestinal conditions (acid, bile, pancreatic enzymes), to colonize the human intestinal mucosa and to exert their beneficial effects, especially by inhibiting potential pathogenic bacteria [87]. Furthermore, other required characteristics include their lack of pathogenicity, the ability to survive during technological processes (fermentation, drying, presence of inhibitors such as salt or nitrite, low water activity, acidic pH) [95,96], the property of not producing biogenic amines and also the absence of specific antibiotics resistance [97,98]. Within the entire range of meat products, fermented dry sausages are the most appropriate for probiotic bacteria incorporation due to their preparation and consumption without heat treatment, thus increasing probiotics chances for surviving [99]. Furthermore, the meat matrix is considered protective for probiotics bacteria during their crossing through the gastrointestinal tract, meanwhile enabling their supply as health benefits [100]. Several studies demonstrated the feasibility of using *Lactobacillus* species as potential probiotics in fermented meat products [99,101,102]. One study regarding the survival capacity of lactic acid bacteria used as starter cultures for meat products showed that *Lactobacillus sakei* and *Pediococcus acidilactici* possess great viability rates under acidic pH conditions and a high concentration of salt [101]. *Lactobacillus plantarum, Lactobacillus casei, Lactobacillus rhamnosus* and *Lactobacillus paracasei* have also been reported as potential probiotics in meat products [102].

Ba et al. [103] studied the effect of the fermenting process and temperature on *Lactobacillus plantarum* applicability as a probiotic in sausages. Three samples of sausage mixture were inoculated with 10^5^ CFU g^−1^ *Lactobacillus plantarum*. The used concentration of inoculated bacteria was in accordance with the one reported in other studies [104,105]. By applying processing procedure at three different temperatures (20, 25 and 30 °C), it was indicated that all the samples inoculated with *Lactobacillus plantarum* presented a much higher *lactobacillus* count comparative to the control non-inoculated sample and suggested that these bacteria could adapt in meat mixtures during fermenting and ripening processes. However, the fermenting temperature considerably affected some technological and sensorial quality traits of the product, such as lipid oxidation level, spoilage bacteria count, biogenic amines level, color and textural profile. The results concluded that the most significant quality improvement of sausages inoculated with *Lactobacillus plantarum* was observed when a temperature of 30 °C was applied [103]. In the case of these products, special attention should be paid to the viability of probiotics, and to the major factors affecting their viability. An equilibrium between the technological challenges and health benefits should also be balanced by the sensory characteristics. On the other hand, probiotic strains proved to be inhibited by some ingredients of meat products (salt, nitrate and nitrite) or by technological conditions during different stages. Some studies have reported poor survival of probiotic microorganisms in fermented meat products [106]. For increasing their survival ability in adverse environments, the microencapsulation has been suggested as a promising method. The technique consists of packaging microorganism cells in polymeric capsules to ensure a protective physical barrier to the living cells [107]. The most-used coating materials for probiotic encapsulation are alginate, starch, glycerol, k-carrageenan, xanthan gum, gelatine, whey proteins, fatty acids and chitosan [108,109].

In the study of Song et al. [110] it is indicated that the encapsulation of *Bifidobacterium longum* in fermented sausages preserved about a half of probiotic bacteria viability in the product, after fermentation and 22 days of maturation. Furthermore, the inoculated product presented the lowest lipid oxidation level, a higher level of unsaturated fatty acids and superior sensorial scores for color, odor and taste, comparative to the non-inoculated control sample.

Another research that studied alginate encapsulated cells of *Lactobacillus plantarum* added in dry-fermented sausages demonstrated that, after 60 days of storage, a higher number of living bacteria (8.34 log CFU g^−1^) and a lower lipid oxidation level (0.602 mg MDA kg^−1^) were highlighted relative to free cell addition of *L. plantarum* in the sausages samples (8.02 log CFU g^−1^, 0.625 mg MDA kg^−1^) [111].

An important aspect of probiotic microorganisms is their ability to produce high yields of bacteriocins, acting like bioprotective agents. The addition of encapsulated *Lactobacillus casei* in fermented sausages induced a high resistance to spoilage and a significant reduction of *pseudomonas*, *enterobacteria* and *staphylococci* bacteria [112]. The addition of *Enterococcus faecalis* free cells to ground beef (4 log CFU g^−1^) inhibits the growth of *Escherichia coli, Clostridium perfringens* and *Listeria monocytogenes* [113], while the encapsulated and free cells of *Lactobacillus reuteri* inactivate *Escherichia coli* O157:H7 pathogenic bacteria and significantly increase the shelf-life of dry fermented sausages [114]. The use of the encapsulated probiotics in the meat products has the main advantage of the preservation of the probiotics’ viability despite the harsh conditions of the technological processing or the acidity of the stomach. However, further studies are necessary to prove that the encapsulated probiotics might exert real health benefits on the host.

## 3. Reducing the Harmful Components

### 3.1. Salt Reduction

The meat industry, as well as the entire food industry, aims to deliver lower salt levels in processed food. The World Health Organization has recommended a reduction in salt dose in adults to less than 5 g/day (the equivalent of 2 g sodium/day) [115]. High sodium intake (with salt being the major contributor) is associated with several health problems, such as osteoporosis, kidney disease, stomach cancer and high blood pressure, which constitute a major factor in the risk of cardiovascular diseases such as heart disease, heart failure and stroke [116]. Sodium chloride possesses an important role in processed meat products, both for sensory and technological purposes [117]. It is widely recognized that salt plays a role in enhancing the texture and flavor of food, providing specific processing characteristics through the water-binding properties and solubilization of myofibrillar proteins (actin and myosin), ensuring at the same time microbiological safety by inhibition of spoilage microorganisms [118,119].

After bread and cereal-based foodstuffs, processed meat products represent the largest source of sodium in the European diet [120]. It is considered that meat products contribute up to 25% of the salt/sodium in the human diet. Thus, the meat industry and researchers have been interested in the development of some strategies for finding salt analogues. These strategies include direct reduction in salt level, complete or partial replacement of salt by low-sodium ingredients, use of flavor enhancers and a combination of previous methods with some innovative technologies (ultrasound, high-pressure processing, pulsed electric field) [121].

One of the simplest possibilities for reducing the level of added salt is the stealthy method. This method involves a gradual reduction, over a long period, of the salt in meat products, so that the consumers will not perceive the changes in the saltiness. However, the method presents some limitations because salt contributes to system preservation and, as a consequence of its reduction, the shelf-life of products is shortened. Furthermore, it requires a long time for implementation and can also negatively affect the palatability of the product. On this line, Delgado-Pando et al. [122] determined the lowest acceptable salt level in bacon and cooked ham, without using other ingredients or additives. Results showed that salt levels could be reduced by up to 34% in bacon and 19% in ham, without affecting the physicochemical, microbiological and sensorial properties of products. In another study, Aaslyng, Vestergaard and Koch reported that moderate salt reduction of the conventional recipe is also possible for ham (from 2.3% to 1.8%) and sausages (from 2.2% to 1.7%), without significantly altering their sensorial properties, shelf-life and safety [123].

An alternative method for the salt reduction in meat products consists of the replacing of sodium chloride with other mineral salts. The most common types of salt substitutes are potassium chloride (KCl), calcium chloride (CaCl_2_), magnesium chloride (MgCl_2_), potassium lactate (KC_3_H_5_O_3_), magnesium sulphate (MgSO_4_) and calcium ascorbate (C_12_H_14_CaO_12_) [124,125]. KCl is the most used NaCl replacer because of its similar behavior regarding inhibition of protease activity, protein solubilization and an equivalent preservative effect [126]. However, potassium chloride can be used at a substitution rate up to 40—50%, because at higher concentrations it reduces saline taste, produces bitter and metallic tastes and generates important flavor defects [127,128]. Some studies showed that sensorial defects of meat products, caused by the replacement of NaCl with KCl, can be reduced by the addition of different flavor enhancers, such as amino acids (lysine, histidine, arginine), disodium inosinate, disodium guanylate [129], yeast extracts, seaweeds, vegetable protein hydrolysate and monosodium glutamate [127]. Thereby, replacement of 50% and 75% NaCl with a mixture of KCl, monosodium glutamate, disodium guanylate, disodium inosinate, lysine and taurine, in fermented cooked sausages and masked the flavor defects caused by sodium chloride reduction [129]. Another study demonstrated that the addition of arginine (1%) and histidine (0.2%), singles or combined, improved considerably sensorial characteristics, texture profile and emulsion stability, caused by the replacement of 60% NaCl with KCl in emulsified meat products [130].

Edible algae, due to their high content of minerals (Na, K, Ca, Mg, P, Mn, I, Fe and Zn), were used in several studies as salt-replacers in reformulated meat products. Moreover, they represent an important source of bioactive compounds and nutrients, such as proteins, peptides, dietary fibers, polyunsaturated fatty acids, phenolic compounds and vitamins, all recognized for their beneficial effects on human health. Thus, Choi et al. [131] concluded that incorporation of edible seaweeds (sea mustard and sea tangle) in frankfurters with 60% reduced salt, confers better color, flavor, juiciness and tenderness to the product comparative to the control sample with regular salt. Fellendorf et al. [132] described similar results by addition of Wakame in low-salt black puddings. According to the authors, reformulated products presented a similar color, but a higher saltiness and spiciness scores than control sample [132]. Meanwhile, some other results must be also taken into consideration. Recent research studied the effect of four edible seaweeds’ incorporation (*Undaria pinnatifida, Porphyra umbilicalis, Himanthalia elongata* and *Palmaria palmata*) in frankfurters with 50% less sodium. Results revealed that the addition of the algae caused darker color, flavor changes and considerable reduction of hardness and chewiness to the product relative to controls, so that it can be concluded that there are negative effects on sensory profile and acceptability of these reformulated meat products [133].

### 3.2. Nitrate and Nitrite Reduction

For decades, sodium nitrite and nitrate have been used in different meat products for their preservative effect, for developing the characteristic color of cured meat, for providing specific flavors or for preventing lipid oxidation [134]. By combination with salt, sodium nitrite becomes an efficient inhibitor for the growth of some anaerobic bacteria, such as *Clostridium botulinum*, which is the source of botulinum toxins and other pathogens like *Bacillus cereus*, *Clostridium perfringens*, *Listeria monocytogenes* or *Staphylococcus aureus* [135,136]. Antimicrobial effects of nitrite are explained by reducing oxygen uptake, breaking the electron transport chain and inactivating some metabolic enzymes [137,138]. Sodium nitrite is also responsible for the development of cured meat color. Added to meat, it is converted to nitrous acid under acidic conditions of muscular tissue. Nitric oxide, formed from nitrous acid, reacts with myoglobin and produces nitroso myoglobin, a dark red-colored pigment. During thermal processing, nitroso myoglobin is converted to nitroso hemochrome, the stable pink color compound. Another property of the nitrite is its ability to reduce lipid oxidation. Nitric oxide reacts with oxygen and reactive oxygen species and stops the lipid autooxidation reactions. Furthermore, nitric oxide binds and stabilizes the iron in heme, limiting its prooxidant activity. The antioxidant effect of nitrite has been reported at levels of up to 40 ppm [137].

Finally, it is well known that nitrite makes an important contribution to the flavor of meat products, even if the mechanism for this effect is not fully understood. Safa, Portanguen and Mirade [139] considered that, due to its inhibitory effect on lipid oxidation, nitrite delays the formation of carbonyl compounds, which are responsible for the rancid flavor. Moreover, Villaverde et al. [140] concluded that nitrite induces a Strecker reaction and the formation of aroma-active aldehydes.

Nitrate and nitrite have different effects on human health. The epidemiological and clinical studies have associated their dietary intake with an increased risk for some diseases [135]. In the first phase, nitrate is reduced to nitrite by endogenous bacteria. Nitrite is an extremely reactive compound, especially under acidic conditions. It can react with several meat constituents, such as amino acids, amines, myoglobin and phenolic compounds. As a nitrosating agent, nitrite reacts with secondary amines and produces potent carcinogenic nitrosamines [141,142]. Moreover, the degradation products of nitrite also react with the heme groups of hemoglobin, reducing blood capacities to transport oxygen to tissues and leading to methemoglobinemia [143]. In contrast, there are some studies which suggest several benefits of nitrite on human health. Dietary nitrites proved to be important sources for endogenous synthesis of nitric oxide in the human body. Recent research related the ability of nitric oxide to control blood pressure, reduce inflammation, improve vascular function and prevent cardiovascular diseases like heart attack, stroke and atherosclerosis [144,145].

Currently, another important challenge for the meat industry is to find solutions for reducing the supplemented nitrate and nitrite in meat products, to decrease nitrite intake [137]. This challenge is even more difficult because nitrite has multiple functions simultaneously, such as the development of characteristic color and flavor, respectively, antimicrobial and antioxidant activity. Alternative compounds or/and technologies which can be used as nitrite substitutes must achieve the quality and safety improvements expected by consumers nowadays, without altering the specific characteristics of processed meat products.

According to Correira et al. [146] and Colla et al. [147], nitrate content in some vegetable species is frequently higher than 2500 mg/kg. A possibility for partial or total replacing of sodium nitrite in meat products consists of using nitrate-rich vegetable extracts [148,149]. The main vegetable used as a nitrate source is celery, but it is considered a major food allergen [150]. Researchers have attempted to use other vegetable sources, such as Swiss chard, spinach, beetroot, radish and leek [151,152]. There are two possibilities for using vegetable extracts in meat processing. The first method consists of the direct addition of plant extracts into the meat mixture or brine, together with a starter culture of nitrate-reducing microorganisms (*Staphylococcus carnosus, Staphylococcus xylosus*) used for conversion to nitrite. This procedure is widely applied, especially for obtaining dry-cured meat products, whose long maturing periods are favorable for nitrate from vegetable extracts to be converted [134,153,154]. The second method involves the addition of pre-fermented or cultured vegetable juice or powder, with measurable nitrite content, after conversion of nitrate during a controlled fermentation process. This method is used in particular for the obtaining of cooked meat products [138]. Both described methods present a great interest and several applications with favorable results are described in the scientific literature.

Ko at al. [155] reported that the addition of young radish in cooked sausages as a natural source of nitrate increased lipid oxidative stability and prevented the growth of *Listeria monocytogenes* and *Staphylococcus aureus*. Furthermore, the color of sausages was comparable to that of the control sample, where sodium nitrite was used. Similar results were obtained by Jeong et al. [156], when radish powder was added to cured pork products, in a concentration of 0.4%. Redness, total pigment and curing efficiency of reformulated sausages were like the traditionally cured control sample. According to Shin et al. [157], incorporation of 2% pre-converted nitrite from Swiss chard powder in cooked pork patties showed higher contents of nitroso-heme pigment and similar microbiological stability relative to control with sodium nitrite added. Sucu and Turp [158] did not identify significant differences in sensory properties of Turkish fermented beef sausages with 0.35% beetroot powder added and a control sample treated with 150 mg/kg sodium nitrite, during 56 days of storage.

### 3.3. Preventing the Formation of N-nitrosamines

The meat products can be contaminated with N-nitrosamines, which are usually formed due to the reaction between nitric oxide, generated from nitrite and secondary amines resulted from protein degradation [159]. N-nitrosodimethylamine (NDMA), N-nitrosodiethylamine (NDEA), N-nitrosopyrrolidine (NPYR) and N-nitrosopiperidine (NPIP) are some of the most common nitrosamines found in meat products [160]. Formation of these compounds represents a complex process which is influenced by several factors like meat composition, nitrite concentration, heat treatment and smoking, decarboxylase activity, pH-value, water activity, presence of precursors (e.g., pyrroperine, pyrrolidine, piperine and piperidine from black pepper), catalysts (Fe(III), but not heme of myoglobin) or inhibitors (e.g., antioxidants) [159,161]. Nitrosamines are relatively stable compounds, but they can be metabolically activated, becoming carcinogenic. Some studies showed that even in low doses, these compounds have a carcinogenic capacity for tumor induction at laboratory animals [136,160].

Besides their recognized antioxidant properties, ascorbate and ascorbic acid also possess a strong *N*-nitrosamines inhibitory property [162]. The mechanism of nitrosamines inhibition by ascorbate is not fully elucidated but might be considered a consequence both of its nitric oxide binding and quantitative reduction of the residual nitrite [135]. Ascorbic acid and its derivates (ascorbate, erytorbate), together with other antioxidants, were widely studied as possible inhibitors of N-nitrosation reaction in the processed meat. Walters et al. [163] concluded that treatment of bacon with up to 300 mg/kg ascorbate, significantly decreased NPYR formation after frying. Recently, Zhou and Wang [164] reported the ability of rosemary extract, grape seed extract and green tea polyphenols to reduce residual nitrite and nitrosamine content in smoked sausages.

## 4. Alternative Technologies

### 4.1. High-Pressure Processing

High-Pressure Processing (HPP) is considered an ecological non-thermal technology, consisting of high pressure treatment applied to the food system, under isostatic conditions [165]. The pressure is uniformly distributed through the product by a liquid transmitter, without being influenced by its size or shape. Usually, HPP parameters involve hydrostatic pressure levels between 300 and 800 MPa, room temperature, and a period between a few seconds and 20 min [166]. HPP has emerged as an alternative to the preservation methods by heat treatment, but recent studies demonstrated its applicability for other purposes. High-Pressure Processing can be applied both to liquid and solid foods to extend their shelf life, maintain nutritional value, improve sensory properties and quality and to develop new and healthier foodstuffs [167]. Meat and meat products are suitable for HPP due to their internal structure and chemical composition (high protein and water contents). Several studies reported the possibility of HPP to improve the tenderness, one of the main sensory attributes of meat. The effect of high-pressure treatment on meat depends both on the processing parameters (pressure, temperature, exposure time) and the rigor stage [168]. It is considered that a pressure value ranging between 100–200 MPa, applied at ambient temperature, leads to an increase in glycolysis and proteolytic activity, thus proving to be favorable for improving the tenderness of pre-rigor meat [169]. On the other hand, the application of HPP to post-rigor meat, at room temperature, showed no changes in tenderness, but only an increase in its toughness proportionally with an increasing pressure level [170]. The toughness enhancement by using HPP is assigned to the compression of myosin filaments on the Z-line of sarcomeres and removing the weak I-band zone [166]. By applying HPP to post-rigor meat, an optimum tenderizing effect proved to be when a pressure of 150–200 MPa and 60–70 °C temperature were used [169].

Several studies concluded that HPP constitutes a possible complementary technology in the strategy of direct salt reduction into meat products. Reformulated pork sausages, prepared with 20% fat and 1% salt, were subjected to a 200 MPa pressure for 5 min, at a temperature of 10 °C. Sensory evaluation indicated no significant differences relative to the untreated control sample. Moreover, HPP improved textural properties and reduced cooking loss of reduced-fat and reduced-salt sausages [171]. Pietrasik et al. [172] investigated the effect of high-pressure treatment on reduced-sodium sausages. Results showed that when a 600 MPa pressure was applied for 3 min, at a low temperature (8 °C), no negative effects on their sensory quality were observed, but only an increase of shelf-life (up to 12 weeks) and water retention capacity.

### 4.2. Cold Plasma

Cold plasma (CP) represents an ionized gas, obtained in conditions of atmospheric or low pressure, which contains a mixture of biologically reactive species, such as positive and negative ions, electrons, photons and free radicals. The type and concentration of these reactive elements depend on several factors, like gas composition, humidity level, characteristics of plasma source (dielectric barrier discharge, corona discharge or atmospheric plasma jet), discharge power and exposure time [173].

Some recent studies have focused on the potential application of cold plasma as non-thermal pasteurization or sterilization methods for different food products, such as meat, cheese, cereals or vegetables [174,175]. Varila, Marcone and Annor [174] described the efficiency of cold plasma technology in extending the shelf-life of meat products, due to its ability to inactivate a wide spectrum of microorganisms, including biofilms, fungi, spores and some viruses. Multiple studies have demonstrated that cold plasma treatment of meat and meat products was efficient for the inactivation of some pathogens like *Staphylococcus aureus* (Beef jerky) [176], *Salmonella enterica* and *Campylobacter jejuni* (Skinless chicken breast and chicken tight) [177], *Escherichia coli* and *Listeria monocytogenes* (Raw pork loin) [178] and *Salmonella typhimurium* (Bacon) [179].

Besides microbial reduction, another application could be the use of cold plasma-treated water as a potential curing agent for meat batter [180]. Previous studies have reported that cold plasma treatment of liquids may lead to nitrite generation, resulting from plasma–water interaction [181]. Jung et al. [180] investigated the influence of direct cold plasma treatment of meat batter during mixing. The results showed a gradual increase in the nitrite level in meat composition by up to 65.96 ppm, after 30 min of plasma treatment. This level of nitrites ensured the development of a specific cured pink color after heating, which confirmed that cold plasma treatment replaces nitrite addition to meat products.

### 4.3. Ultrasound

Ultrasound (US) is considered a non-thermal, emerging technology with great potential and wide application possibilities in the food industry. US uses sound waves with higher frequencies than the human audible limit, and its application field in food processing is possible at frequencies between 20–100 kHz [182]. When US waves are propagated through a medium, they cause compression and rarefaction of its particles, inducing the cavitation phenomenon. Cavitation generates a large number of microscopic bubbles, which become unstable and collapse after consecutive cycles of ultrasound waves, producing high local temperatures and pressures. Furthermore, the implosion of cavitation bubbles generates high-speed microjets (100–340 m/s), which can induce physical disruptions in the food matrix [182,183]. These occurrences generate changes in biological materials (cell membrane disruption, protein structure alteration, emulsion generation and chemical reactions), which underlie their application in food processing. Recent studies indicated the potential application of high-intensity ultrasound in meat systems, mainly in salting, tenderizing, cooking, homogenization and microbial control [183]. The influence of US on the formation and stability of meat emulsion was also reported. In their study, Cichosky et al. [184] demonstrated that sonication (25 kHz, 60% amplitude, 5.5 min) exerts positive effects on meat emulsion quality. Results highlighted better stability and improved texture parameters of the emulsion (chewiness, cohesiveness and hardness), without a negative impact on proteins and lipid oxidation. Other researchers focused on the US application as a strategy to reduce salt and phosphate added to content in meat products, since it is known that sonication increases mass transfer processes in liquid–solid systems [185]. In their study, Barretto et al. [186] evaluated the effects of NaCl reduction and ultrasound treatment (20 kHz, 600 W/cm^2^, 10 min) on cooked pork ham. The authors confirmed that application of ultrasound improved the texture and allowed a 32% reduction of sodium content in cooked ham. Recently, application of US (25 kHz, 60% amplitude, 20 min) on meat emulsions prepared with basic electrolyzed water showed the possibility to reduce up to 30% of sodium chloride, without decreasing technological quality [187].

## 5. Conclusions

Numerous strategies for obtaining healthier meat products have been developed in the last few years. Individually or in an applied combination, several methods have shown the possibility of obtaining improved meat products in terms of nutritive value, sensory characteristics and preservability. Fruits, vegetables, by-products and other different plant materials can constitute a good alternative and an inexpensive source of bioactive compounds, such as antioxidants and dietary fibers. Antioxidant properties to reduce proteins and lipids degradation, to preserve color and to inhibit the formation of toxins in meat products are in addition to their ability to reduce oxidative stress. Incorporation of dietary fibers improves physicochemical properties of meat products, these compounds being at the same time helpful ingredients in the prevention of nutritional and diet-related disorders. Fermented meat products can represent a proper vehicle for probiotic microorganisms, recognized for their positive effects on many human diseases. For reducing salt, nitrate and nitrite quantities added to meat product composition until acceptable values and to prevent known harmful effects, some alternative compounds and technologies have proved to have efficiency in their partial substitution, with no alteration of their specific characteristics. Alternative technologies like HPP, UF and CP, which are non-thermal processes, can generate multiple advantages in obtaining improved meat products. Thus, they can enhance safety and extend the shelf-life of products by their antimicrobial effect, improve texture or tenderness of meat and meat products, allow reducing technological process and also significant energy economy.

As a final conclusion, it can be stated that some of the studies presented in this review were performed only on a laboratory scale and proved the efficiency for several methods so that this research needs to be transferred to the meat industry in the near future. It would also be advisable that upcoming studies analyze the effects of a combined use of these methods in order to obtain meat products with high acceptability by nutritionists and consumers.

## Figures and Tables

**Table 1:** Effects of dietary fibers on the properties of various meat products.

Developed Product	Fiber Source	Recommended Dose (%)	Effect on Meat Product Quality	**Reference**
Beef patties	Rice bran	2; 4; 6	Substitution of fat and total trans fatty acids.	[24]
Pork and beef sausage	Amorphous cellulose fibers from the husk of oat, soy and rice grains	1;3	50% fat reduction; Increasing emulsion stability and consistency.	[25]
Chicken nuggets	Chia flour	10	Decreasing the moisture, saturated and monounsaturated fatty acids contents; Increasing the total amount of dietary fibers.	[26]
Emulsion type sausages	Inulin	6	Reduction of fat and energy content; Sensory acceptance is comparable with the one of a traditional product	[27]
Meatballs	Rye bran	20	Reduction of total trans fatty acids; Reduction of weight losses, improving nutritional value, health benefits and color.	[28]
Frankfurter sausages	Citrus fibers Rice bran	1.5 0.5	Positive effect on the acceptability; Adequate hardness and cohesivity; Acceptable decrease of color intensity.	[29]
Cured Bologna sausage	Citrus fibers Cherry powder	1 0.3	Replacement of fat and sodium tripolyphosphate; Hardness improvement.	[30]

**Table 2:** Effect of natural antioxidants in different types of meat products.

Developed Product	Antioxidant Source	Recommended Dose	Effect on Meat Product Quality	Reference
Beef burgers	Rosemary extract	0.5%	Oxidative stability and sensorial characteristics preservation of burgers stored in freezing at −18 °C for 30 days.	[35]
Frankfurters sausages	Strawberry extract	130–350 mg GAE/kg	Reduced lipid oxidation during 30 days of 4 °C storage.	[36]
Dry-cured sausages	Grape seed	50; 200; 1000 mg/kg	Suppression of lipids oxidation during ripening and storage periods.	[37]
Raw pork patties	Guarana seed	250; 500; 1000 mg/kg	Reduction of carbonyls and TBARS formation.	[38]
Pork sausages	Banana inflorescences	0.5; 1; 1.5; 2%	Positive effect on the control of lipids oxidation during storage; Sensory acceptance unaffected even when 2% dose was used.	[39]
Minced meat	α137–141peptide from hydrolyzed bovine hemoglobin	0.1; 0.5%	Inhibition of lipids oxidation at the same level as BHT synthetic antioxidant.	[40]
Chicken products	Protein hydrolysates from tea residues	0.1; 0.5; 1%	Strong antioxidant effect, similar to BHT synthetic antioxidant.	[41]
Homogenized ground beef	Casein calcium peptides	2%	Inhibition of about 70% lipid oxidation	[42]
Pork patties	Whey bioactive peptide	2%	Inhibition of oxidative deterioration during storage.	[43]

**Table 3:** Antioxidant dietary fibers in different meat products.

Developed Product	ADFs Source	ADFs Level (%)	Effect on Meat Product Quality	Reference
Chicken hamburgers	Red grape pomace	0.5; 1.0; 1.5; 2.0	Improved color; Inhibited and retarded lipid oxidation; No adverse influence on sensory attributes.	[58]
Sheep meat nuggets	Guava	0.5; 1.0	Increased dietary fibers and phenolics content; Improved oxidative stability; No change in textural properties; No adverse effect on sensory properties.	[59]
Cooked sausages (bolognas) and dry-cured sausages	Lemon albedo	2.5; 5.0; 7.5; 10.0	Increased dietary fiber amount; Decreased residual nitrite; Increased hardness; Better sensory scores.	[31]
Cooked sausages (bolognas)	Orange fiber powder (0.5, 1.0, 1.5 and 2.0%)	0.5; 1.0; 1.5; 2.0	Intensified color of product; Increased dietary fiber content; Increased hardness; Less elasticity than control product.	[60]
Functional mutton patties	Cabbage powder	6.0	Inhibition of lipid oxidation; Better sensory scores; Improved textural properties; Increased nutritive value.	[49]
Spent hen nuggets	Gooseberry pulp powder Seed coat	0.5 1.5	Improved shelf-life; Improved physico-chemical properties; Better acceptability of product.	[61]
Low-salt beef patties (raw and cooked)	Wakame seaweed	3.0	Improved water-binding properties; High antioxidant activity; Improved textural properties; No adverse effect on product acceptability.	[62]
Frankfurters	Walnut	25	Increased polyunsaturated fatty acids amount; Increased dietary fiber content; Healthier amino acid profile; Improved yield.	[63]
Pork and turkey sausages (Vienna type)	Pineapple pomace	2.5; 5; 7.5; 10	Increased dietary fiber content; Improved color; Decreased values of shrinkage and shear forces.	[54]
Chicken nuggets	Dragon fruit peel	1.5; 3.0	Improved emulsion stability; Decreased lipid oxidation; Improved redness of nuggets; Decreased hardness and gumminess compared to control product.	[64]
Ham pâté	Kiwi fruit skin flour	0.5; 1.0; 2.0	Increased dietary fiber content; Enhanced odor and flavor; Best acceptability at 1% level.	[65]

**Table 4:** Main probiotic microorganisms.

Genus	Species
*Lactobacillus*	*L. acidophilus* [74]; *L. delbrueckii* subsp*. bulgaricus* [66]; *L. brevis, L. fermentum* [75]; *L. casei Zhang* [76,77]; *L.reuteri* [78]; *L. paracasei* [79]; *L. rhamnosus* [80,81]; *L. gasseri* [82]; *L. plantarum* [83,84]; *L. casei* [85]
*Bifidobacterium*	*B. infantis; B. animalis* subsp*. lactis; B. bifidum; B. breve; B. longum* [82,86]
*Saccharomyces*	*S. boulardii* [87]
*Lactococcus*	*L. lactis* [88]
*Enterocccocus*	*E. durans; E. faecium* [89]
*Streptococcus*	*S. termophilus* [90]
*Pediococcus*	*P. acidilactici* [91]
*Leuconostoc*	*L. mesenteroides* [92]
*Bacillus*	*B. coagulans* [93]; *B. subtilis* [94]
*Escherichia*	*E. coli* Nissle 1917 [88]
